# Abstracts and Gleanings

**Published:** 1884-09-20

**Authors:** 


### ABSTRACTS AND GLEANINGS.

**Summer Complaint in Children.**—By "summer complaint" proper is understood a condition to describe which several terms have been employed. Among them we have, as the most common, cholera infantum, follicular enteritis and gastro-enteric catarrh. The diarrhœa, in excess of the natural looseness during the period of dentition, is usually the initial symptom. In many casses of sudden onset the first indication of the disease is vomiting, although this symptom usually appears after the diarhœa has continued for several days. In cases of the more gradual development of the disease, which is, perhaps, the more common form, the stools attain the condition of fluidity only after five or six passages, and their odor is unusually offensive When the watery stools have been thoroughly established, this odor and also the normal odor of the evacuations disappears. The serum of the stools soaks readily into the napkins, leaving the shreds of mucous, and greenish matter, and undigested milk, with which it is loaded, lying on the surface. This greenish matter has been the subject of no little study, and various theories have been advanced to account for it. Lehmann and Merklein have shown that when following the exhibition of calomel, which is so often given in this affection, it may consist of the sulphide of mercury formed in the intestines. Golding Bird, however, demonstrated, in cases in which no calomel or other mercurial had been given, that the color was due to the presence of altered blood. The presence of biliary pigment has also been alleged by some to cause the color, but Hartshorne is disposed, from a weighing of the evidence, to attach most importance to the theory of Golding Bird, in such cases, we presume, as have not been treated with mercurials.

The temperature in summer complaint, during the earlier stages, is usually elevated, the head and abdomen feeling particularly hot to the touch. As the disease progresses apyrexia and a positive subnormal temperature may supervene. In very severe and rapidly fatal cases, however, no elevation of temperature whatever has been discoverable. Emaciation commences with the onset of the disease and progresses apace, one or two days being often sufficient to reduce the plump little infant, who had been the picture of health, to a pale, flabby condition, with hollow, lack-lustre eyes and hollow cheeks. Prostration is also sudden in its appearance and persistent throughout, the little one being apparently incapable of the slightest exertion. There is usually much restlessness, the infant tossing from side to side while it has strength to do so, and utteriug whining cries.

The nervous symptoms in summer complaint are of a serious nature, and, it is to be feared, receive too little attention from the therapeutist, who is wont to direct his remedies more exclusively to the relief of the vomiting and diarrhœa. We do not believe that any treatment which ignores the nervous element in these cases can claim to be successful, and the authors who have written on the subject have, in our opinion, attached too little importance to it; some of them, indeed, do not seem to have recognized the slightest involvement of the nervous system. That the stupor, convulsions and delirium which frequently supervene in severe cases should not have directed more attention to the nervous system seems almost incredible. It was Marshall Hall who first pointed out the fact that many cerebral symptoms, which up to his time had been referred to inflammation or hyperæmia of the brain, are dependent on anæmia alone. To the group of symptoms having this common cause he gave the name hydrocephaloid, from their resemblance to the symptoms of true hydrocephalus. He showed that with children not only repeated losses of blood, but also attacks of cholera infantum, may produce this condition, and suppuration also, but more rarely. But short of these more-developed symptoms we have the nervous irritability, the tossing of the head and the half-closed eyes during sleep, which are such constant symptoms in severe cases, all pointing to the nervous system as the seat of disturbance. Whether this disturbance of the nervous system is central or reflex we are not prepared to state. It is probable, however, primarily reflex, the nerves of the gastric and intestinal parietes becoming irritated to a high degree by the action of the fermenting food, thus giving rise, directly, to excessive peristaltic action of the intestines and vomiting. The nervous system of a child is naturally very susceptible to impressions, and is rendered more so by the disturbing and enervating effect of the summer heat. The fact, moreover, that post mortem examinations do not always reveal inflammatory action as having existed in the bowel might also be adduced in support of the nervous theory of the disease. Huguenin and Bednar lay stress on the fact of the frequent association of brain symptoms, even purulent meningitis, with cholera infantum, but fail to state whether these symptoms are due to reflex action or to the effect of septic matter carried directly to the meninges.

The frequent occurrence of cholera infantum in teething children has, very naturally, led to the supposition of a causative connection between dentition and the disease. It is probable, however, that the only connection of this nature exists in the hyperæsthetic state caused by the irritation of the coming tooth. Unripe fruit is sometimes urged as a cause, but the fact that the disease attacks children who have never tasted such fruit and that the majority, indeed, of those who are attacked have never touched it, disposes of this. The disease is peculiarly one of the city, and the vast majority of those attacked are less than eighteen months of age. While all children of this age are liable to attack, those who are bottle-fed are vastly more liable than those who are reared at the breast. Is there not in these facts the broadest suggestion of the cause? In the country, with its purer air and fresher food, particularly milk, the bottle-fed child is immensely more likely to survive than the similarly fed child in the city. The vitiated atmostphere of the city and the decomposing and fermenting food of the same, carted many miles through the great heat from the source of supply, are, unquestionably, the main factors in the etiology, the depressing influence of the heat and the predisposition caused by detention placing the child in a condition making it peculiarly susceptible to these causes.

**A Year's Experience in Tracheotomy.**—George W. Gay, M. D., writes, in the Boston Medical and Surgical Journal : During the year 1883 I performed tracheotomy twenty-one times for croup. Eleven patients recovered. All but one, a fatal case, were treated in the City Hospital. The cases were not selected, every one coming under our charge being operated upon if requiring it.

Many of the patients had diphtheritic croup, a few membranous, and, occasionally, it was not easy to make an exact diagnosis. Cases presenting enlarged glands and a nasal discharge early in the disease were undoubtedly diphtheritic. On the contrary, cases beginning as an ordinary cold, with no membrane visible in the fauces, no septic symptoms, but having a severe and constant dyspnea, were called membranous croup. It is not of the utmost importance that much time be spent in discussing the difference between the two varieties of croup, considering the fact that both demand essentially the same treatment. Suffice it to say that all of the cases presented severe and continued dyspnœa, due to an acute laryngeal obstruction from one to five days' duration.

One patient was twenty-four years old (died) ; the ages of the others varied from eleven months to nine years : a majority were four or five years old. The youngest who recovered was three.

The duration of the disease at the time of the operation ranged from one to eight days ; the dyspnœa from one to five days. As a rule the shorter the period of obstructed respiration the more favorable the result.

No ether was used in eight cases, and only a few whiffs in the others, merely enough being given to partially control the struggling and fright. Generally the patient had rallied from the anæsthetic before the tube was secured in its place.

Two children died of shock and septicemia a few hours after the operation; the other fatal cases survived from two to five days. None died from hemorrhage. Death resulted from either bronchitis or blood-poisoning. Every case but one derived more or less temporary relief from opening the trachea, and, so far as I know, no life was shortened by the operation. The upper rings of the trachea were usually incised, and also the isthmus of the thyroid, if necessary. In a baby lately operated on at the age of nine months the cricoid cartilage was divided with the result of greatly facilitating the introduction of the tube.

Venous hemorrhage was quite free in many cases, but no trouble was ever experienced from blood getting into the bronchi. By inserting a tenaculum or hook into the trachea just below the cricoid cartilage and lifting it up the windpipe is under control, and it is not necessary that the rings be exposed before they are divided. At all events I havs not found it to be so in many of my later operations. Beginners, however, had better see the rings before they cut them. The tube having been secured by tape, a piece of cotton flannel spread with cosmoline is placed between the plate and the skin to prevent irritation.

*After Treatment:* Milk, ice-cream, and beef tea were the favorite articles of food. Nourishment was also administered by the rectum. Alcohol was never given unless the patient exhibited symptoms of marked exhaustion, when champagne was added to the diet. Several of the successful cases received no liquor during the treatment. Quinine and aromatic spirits of ammonia were given in every instance, while iron and chlorate of potash were not resorted to.

Next to nourishment I consider *steam* to be the most important part of the treatment. It is conducted from the radiator through a rubber tube, and directed upon the neck of the patient. The vapor is warm, moist, and does not condense in sufficient quantity to saturate the clothing. Atomized or medicated liquids are not used at present. Lime-water often produced a disagreeable erythema of the face, and thinking that possibly it might act as an irritant to the air passages, pure steam was substituted, and so far it seems to act as favorably as did any of the sprays formerly in vogue.

In all cases the patient received steam half the time, while to the more serious it was constantly supplied. The very great benefit derived from breathing the warm vapors was demonstrated beyond a doubt in many instances. Under its use the secretion would soften, the respiration would become easier, the child would become quiet and fall asleep. The importance of a constant and generous supply of steam can not be overestimated in this affection.

In the favorable cases the tube was worn from six to fifteen days, the average time being nine days and a half. I have found the most satisfactory way of getting rid of the tube to be as follows: At the end of a week, if the respiration is free, the tube is taken out quietly, and the child is let alone. No trials are made to see if he can breathe through his mouth. As the tracheal wound contracts natural breathing through the larynx is gradually restored. With one exception this plan has worked well. In the case of a little girl, after the tube had been taken out, occasional attacks of dyspnœa would come on, which were relieved by the nurse's opening the wound with the dilator and turning on more steam. The child soon learned to call for this instrument whenever she felt an attack approaching. The use of the tube was not again resorted to, and in a few days the dyspnœa ceased, and the child recovered.

I cannot close this paper without calling attention to the importance of having intelligent, skillful and devoted nurses in charge of these patients. Two sets are necessary, one for the day and another for the night, and they should have received special instruction in taking care of the tube, and also in removing or replacing it in an emergency. I can not but feel that my success during the past year was due in no small measure to the admirable care which the patients received from the nurses of the hospital training school.—*Louisville Medical News.*

**Burns and Scalds.**—Dr. G. O. Smith, in Medical Summary, says: Hoping to benefit some sufferers and especially to assuage the sufferings of childhood, I will report two cases of burns and scalds treated by glycerine.

Case first was my own child, who burnt her hand against the stove. Not having any sweet oil at hand, I substituted glycerine. Almost instantly the crying and screaming ceased. I was surprised. I had never read of glycerine for burns. Soon after I burned my own hand and again used the glycerine. The severe smarting and burning immediately ceased. I began to think I had made a discovery of much value.

Case 2. On January last was called to see Anna C., aged twenty-two months, who had, unobserved, seized the handle of a spider that contained meat parboiling on a hot stove. The scalding hot water came pouring on the throat, breast, hands and arms. I was called in haste. Found the child in great pain. Applied glycerine to the scalded surface, when the child ceased its screams and was comparatively easy. Case made a good recovery. The glycerine should be pure and entirely free from oxalic or formic acids, else it acts as a positive irritant. The dressing is cleanly and does not dry and stick like other dressings. After a few hours treatment with the glycerine to remove all pain and to extract the fire, I then paint the burnt spots with pure linseed oil.

**The Water-Cure in Diphtheria.**—Dr. Gasparini (Gazz. Ital. Lomb., February 16, 1884,) accepts Morell Mackenzie's definition of diphtheria, and looks upon it as an acute infectious general disease, with a tendency to the production of certain local manifestations. His treatment consists in wrapping the patient in a cold wet sheet, repeating the packing three or four times a day, according to the height of the fever. Cold compresses are to be kept continually to the throat. He also uses gargles of the alkaline sulphites, carbolic acid, etc., as disinfectants, never caustics. In 1875 six cases were thus treated; all recovered, the disease lasting from five to eleven days. In 1879 seven cases recovered under the same treatment, the average duration of the disease being ten days. This treatment is well borne, and the patients like it; its antipyretic action is marked, though transitory. Stimulants at the same time are to be freely administered. The duration of the disease is not influenced; but convalescence is shortened and the strength is more quickly recovered.—*Med. and Surg. Rep.*

**Syrupus Acidi Hydriodici.**—[Syrup of Iodide of Hydrogen.]—Dr. Craig writes to the Medical and Surgical Reporter: During an experience of several years, I have thoroughly tested this preparation in acute inflammatory rheumatism, chronic bronchitis, and scrofula. In the first disease I prescribe for adults from two to three teaspoonfuls every two or three hours, in a wineglass of water, during the febrile stage, until relieved; this, in my experience, has always occurred within forty-eight hours. I then reduce the dose to one teaspoonful, and continue for five or six days at longer intervals. I have never observed any heart complications under this treatment, the remedy preventing exudation and organization of plastic material.

In chronic bronchitis, its effects have been very gratifying, not only affording prompt relief to the cough, but also to the secondary symptoms of asthma, which are so apt to occur in these cases. I prescribe from one to two teaspoonfuls three times a day, in water.

In scrofula and other cases requiring the alterative action of iodine, a teaspoonful three times a day, in water, will be found to act much more satisfactorily than potassium iodide, as it agrees with the stomach and does not disturb the appetite; if anything, it increases it, and instead of depleting the system seems to possess tonic properties.

It acts admirably in syphilis; in combination with biniodide of mercury, its use can be continued for a long time without any unpleasant effects.

I would also speak of the success and peculiar fitness of this remedy in lead poisoning and paralysis resulting therefrom, as it is the true theoretical antidote to this condition, converting the lead in the system into its iodide, which, owing to its solubility, is readily eliminated through the various excretory channels.

The non-irritant character of this preparation, its prompt action and its palatability very strongly recommend it to favor.

The syrup I have used is that prepared by R. W. Gardner, of New York.

**Lung Extirpation.**—Sir Spencer Wells has written to a medical journal concerning the important matter of operating upon, or removing, diseased lungs or portions of them. He believes that surgeons should prepare to meet these operations by practice upon the cadaver and, if need be, upon living animals. In speaking of the remarkable experiments in lung extirpation by Dr. Biondi, of Naples, Sir Spencer says: "Of sixty-six operations on sixty-three animals, 36 were followed by recovery; of fifty-seven where one entire lung was removed, thirty recovered; and in the six cases where the apices or only one lobe were removed, all recovered.." —*National Druggist.*

**Cases of Collapse and Sinking.**—It is well to know that in cases of collapse and sinking injection of thirty minims of ether into the skin, after the common fashion of injecting morphia, is a most valuable and successful means of restoration. The action of ether in such a case is speedy and effectual. Such a hint is useful to all who, away from medical aid, may, under any circumstances, require to exercise knowledge in the saving of life.—*N. Y. Med. Times.*

**Success Under Difficulties.**—The famous occulist, Prof. Ludwig Mauthner, of Vienna, has just made a successful operation on the eye of a colleague of his, aged 96 years. A former experiment gave back eyesight to a poor man who had completed his 102d year. Considering the age of these patients, these cases may well be cited as unique in the annals of ocular therapeutics.—*Boston Advertiser.*

**Cardiac Dropsy.**—*Digitalis.*—Although the diuretic action of digitalis in cardiac disease with dropsy is perfectly well known, it is very rarely indeed prescribed in this part of the country in that form in which its diuretic action is most marked. My attention was drawn to this as long ago as fifteen years since by the following case: A man with extreme anasarca from cardiac disease, but without as yet the appearance of the skin or the legs indicative of threatened dermatitis with sloughing, passed several days and nights sitting on the edge of the bed suffering from extreme dyspnœa. Southey's capillary tubes were not then invented, and I always punctured the legs with a needle in cardiac dropsy with great reluctance. I ordered the patient to drink freely of freshly prepared, but weak digitalis. This caused a very copious secretion of urine, which continued until the dropsy had entirely disappeared, and the man actually resumed his occupation of travelling for orders in some business, and I saw him about in the streets occasionally for several years. From that time I have invariably used this treatment, and some of the cases have been very remarkable. I generally order half a small or medium-sized leaf to be infused, with the addition of a little black tea, in about twelve ounces of boiling water, and taken daily. By this means we get the full diuretic effect of the drug, in addition to its action on the muscle of the heart. The action of the ordinary tincture and of the powder given in pill is, as a diuretic, hardly noticeable, and the usual combination with squills, solution of acetate of ammonia and spirit of nitric ether is much inferior to the infusion. A few months ago I saw, with a gentleman in Leeds, a case of cardiac dropsy very similar to the one related, and his first words when we were alone were, "Well, I suppose you will say that it is a case in which no good can be done?" and he was apparently surprised at my reply, "On the contrary, I think it is a very promising case." I heard several weeks afterwards that that patient was getting better, or was very much relieved. The fact is, there are many cases of cardiac disease in which a complete breakdown would not have occurred but for some external circumstnces—I mean external to the heart—as an attack of bronchitis or impairment of the health by want of rest, especially if combined with food that is poor in quality; and when dropsy has once occurred, the circulation becomes more and more embarrassed. Let, however, the dropsy be removed, and another chance is given to the heart to recover itself. In this it is aided by the action of the drug on its muscular fibre and by the absolute rest of the patient. Once a year, during some country journey in autumn, I collect a quantity of leaves and dry them for use. The matter is by no means new; but I have taken the opportunity of asking various medical men about their use of digitalis, and have only met with one who uses it in this infusion.—*Dr. James Braithwaite, Lancet, Dec. 17, p. 855.*

**Cholera.**—In an aggravated attack of this disease it is almost impossible to rely upon oral and anal medication because of the continued vomiting and purging; beside, the admixture of remedies with the profusely eliminated serum and accompanying enervation of the absorbents forbids the hope of prompt relief. It would, therefore, seem essential that remedies should be introduced hypodermatically and in the worst cases by intra-venous injection. The hypodermatic injection of morphia, it is claimed, has done more for the relief of the disease than any other remedy, and for reaction from collapse atropine similarly administered has been found most reliable. For years, in the Indies, the collapse consequent upon the bite of venomous serpents, even that of the cobra, has been overcome by the intra-venous injection of ammonia, and it would seem, reasoning by analogy, that the treatment of Asiatic cholera should be directly to the circulation, or with only the blood vessel wall between the remedies and the fluid necessary to be influenced. Therefore, a rational plan of treatment would be to combine with some powerful germitoxic, as corrosive sublimate or permanganate of potash, appropriate quantities of atropine and strychnia for intra-venous or hypodermatic medication. By some such combination we could reasonably anticipate the destruction of germ life, both in the blood and wherever it is transmitted, while the atropine would induce reaction, assisted by the strychnia as a stimulant to the nerve centres. If ever the advanced stage of cholera is rendered amenable to remedial agents, it will be when they are introduced so as to produce almost immediate effects.—*St. Louis Med. and Sur. Journal.*

**Two Signs of True Convalescence in Enteric Fever.**—In a communication to the Clinical Society of Paris, Dr. Chauffard indicates as sure signs of true convalescence in enteric fever the occurrence of multiple abscesses and a critical diuresis. The abscesses have a rapid and insidious development, and when once opened they cease to secrete, their walls uniting in a day or two. The diuresis is sudden, and the quantity of urine passed is very large. Dr. Chauffard's observations extend over the past two years, and in no case has he seen a relapse where these signs presented themselves.—*N. Y. Med. Times.*

**Phenic Acid in Yellow Fever.**—Dr. De Lacaille, of Rio de Janeiro, professes to have cured thirty-eight consecutive cases of yellow fever by the use of Declat's preparations of phenic and sulpho-phenic acids, and in grave cases the phenate of ammonium. In the early stages he gives the remedies by the mouth, but in the advanced stages the hypodermic method is necessary. He contrasts very favorably his recent experience with his former sad failures without these drugs.—*N. Y. Med. Times.*

**An Instant Remedy for Poisoning.**—If a person swallows any poison whatever, or has fallen into convulsions from having overloaded his stomach, an instantaneous remedy is a heaping teaspoonful of common salt and as much ground mustard stirred rapidly in a teacup of water, warm or cold, and swallowed instantly. It is scarcely down before it begins to come up, bringing with it the remaining contents of the stomach; and less there be any remnant of poison, however, let the white of an egg or a teacap of strong coffee be swallowed as soon as the stomach is quiet; because these very common articles nullify a number of virulent poisons.—*Exchange.*

**Nux Vomica as a G lactagogue.**—Dr. Posada Arango speaks very highly of the good effect of nux vomica as a stimulant to the secretion of milk. He gives ten drops of the tincture three times a day, and explains its galactagogue properties by its action on the mammary gland, exciting it to secretion, and by its stimulating action on the stomach, facilitating digestion. He recommends strychnia in recent cases of complete suppression of the secretion.—*London Medical Record.—Ibid.*

**The Thermo-Cautery in the Treatment of Anal Fistule.**—Dr. E. Farcy recommends the thermo-cautery in the treatment of fistula in ano, and draws the following conclusions as to its advantages:

1. The operation is rapidly performed, and several fistulous tracts may be operated at the same time.

2. Chloroform is unnecessary, and there is no danger of either primary or secondary hemorrhage, as by other methods.

3. By this method the vitality of the tissues is excited and there is only moderate suppuration. The wound is protected from the direct influence of the air before granulation sets in.

4. There is no fever, no erysipelas, no phlegmonous or purulent infection, no relapse, and the cicatrix is linear. It is therefore the best method for operating in these cases.—*Jour. Pharm.—Ibid.*

**Our Growing Longevity.**—The increasing knowledge of the laws of sanitation and personal hygiene are yearly adding to the average of human life. In an address before the late international health exhibition at London, Sir. James Paget referred to this matter in his usual pointed and telling manner. He indicated as the reasons for increasing longevity and the less amount of sickness, as compared with by-gone times, the fact that there is less intemperance and less immorality now than formerly. We have better, cheaper and more various food, far more and cheaper clothing, far more and healthier recreations; we have, on the whole, better houses and better drains, better water and air, better ways of using them. The care and skill with which the sick are treated in hospitals, infirmaries and even in private houses are far greater than they were. The improvement and extention of nursing are more than can be described. The care which the rich bestow upon the poor whom they visit in their own homes is every day saving health and life; and even more effectual than any of this is the work done by the medical officers of health and all the sanitary authorities now active and influential in all parts of the kingdom. What we want, forcibly insisted the learned lecturer, in conclusion, is more ambition for health, and a personal ambition for renown in health as keen as that is for bravery or for beauty, or success in our athletic games and field sports.—*Medical Age.*

**Flatulence.**—Touching the proper diet, etc., in flatulent dyspepsia, Dr. Bartholow (in College and Clinical Record) says: "We should provide nothing which will contribute to the evolution of carbonic acid gas. A great deal could be accomplished by restricting the diet to the most elementary constituents. What is the elementary diet? It is that provided for the infant during the earliest period of life. It is milk. This patient should at once adopt an exclusively milk aliment; and, in order to render its digestion more easy, the cream should be removed. It should be skimmed once. To insure its digestion, one-fourth the quantity of lime-water should be added, and it should be given every three hours, for this is about the time required for the digestion of milk. About a gill will be taken on each occasion. A patient may well subsist on milk, as it contains all the constituents necessary for the support of the human body; but living on an exclusive milk diet is not an enjoyable existence. After a few days there is a great desire for solid food; there is a feeling of weakness or "goneness," and there is usually constipation. Notwithstanding these disadvantages, the patient should be encouraged to persist in the use of the milk.

"How long should it be continued? The proper rule is to continue the milk until the symptoms for which it has been prescribed disappear. That may be one, two, three, or even more, weeks.

"What else should be done? After this course of milk, which should be exclusive, the patient taking no other aliment, and, in fact, no other drink, we shall add to the dietary such articles as are suited to her condition. Beef tea, made by mechanical process, and white of egg could then be used. The yolk of egg should be avoided, as it contains fatty and other constituents which are difficult to digest.

"What remedies should be given? It is necessary to give remedies which will relieve the gastralgia and at the same time prevent the fermentation of certain constituents of food Creasote is one of the best remedies for this purpose. It may be combined with bismuth and glycerine. It has been lately shown that glycerine has a decided power to prevent fermentation in the stomach, and thus prevent the subsequent distention due to the evolution of gas. I will prescribe the subcarbonate of bismuth, which is better than the subnitrate. The prescription will then be:

| | | |
|---|---|---|
| ℞ | Creasoti | *m* viij, |
| | Bismuthi subcarb | ʒ ij, |
| | Glycerini, | |
| | Aquæ menthæ pip | aa, ℥ j. M. |

SIG.—To be well shaken and a teaspoonful given every three, four, five or six hours, according to the persistence of the pain.

"This patient suffers from an extreme degree of constipation, and under the milk diet this symptom will be greatly increased. As a rule, in such a case it is better to relieve constipation by the rectal administration of remedies. Sometimes a saline does very well; a bottle of Congress or Pullna water or a little Epsom salts will be sufficient. Better than this, as I have just remarked, is an enema, at night, of half an ounce to an ounce of linseed oil, allowed to remain all night and followed in the morning by an enema of soapsuds. Instead of linseed oil, we may use an enema of castor oil suspended in mucilage.

Another remedy which is very efficacious in these cases, and which may be given in the prescription already mentioned, or separately, is arsenic. It has been found that in gastralgia and abnormal fermentation Fowler's solution of arsenic is exceedingly useful. It should be given in small doses, as one or two drops, thiee or four times a day.

**Blindness from Ophthalmia Neonatorum.**—At its last meeting the Ophthalmological Society dealt with a suject of very great importance. It will be remembered that a few months back a committee was appointed to inquire into the prevalence of blindness resulting from a preventable malady—ophthalmia neonatorum. In answer to a very large number of inquiries from private persons, ophthalmic and lying-in hospitals, and from institutions for the blind, the committee has received twenty-three statistical replies, only four of which are considered to be sufficient and trustworthy. In the Belfast Deaf, Dumb and Blind Institution, 30 per cent. of the persons concerned owe their blindness to ophthalmia neonatorum; the London Society for Teaching the Blind to Read gives about the same per centage; the Blind School at York about 40 per cent.; and that at Hull about 35 per cent. These numbers substantially agree with those of foreign investigators, notably those of Reindard, who, on investigation of twenty-two German blind asylums, found 658 blind from this disease among a total of 2,165, equal to 30.5 per cent.—*Lancet.*

**Oleate of Chloral Comp for Pruritus.**—The writer has had the above compound prepared for pruritus ani, the itching of eczema and all other similar cases in which an itching exists which it is deemed expedient to allay temporarily until the means employed for permanent relief act. The compound is made by mixing together one-half drachm each of camphor and chloral and adding to this one ounce of oleic acid. This makes a clear brown liquid having the odor of camphor and it may be scented to suit the taste of the patient.

Messrs. Parke, Davis and Co. made a very satisfactory preparation for the writer and it has been found to answer its purpose admirably. We would request all physicians, having an opportunity to do so, to employ this remedy and report their experience with it. It is a local anæsthetic to the ends of cutaneous nerves and will act as such, its efficacy depending upon the strength in which it is employed.—*St. Louis Medical Journal.*

**Death from Chlorate of Potassium.**—A man, forty-nine years of age, by mistake took a teaspoonful of chlorate of potassium in water every two hours until he had taken, in thirty-six hours, nearly two ounces. Dr. Bohn (who reported the case in the Deutsche Med. Woch.) found him in a condition of collapse, suffering greatly from pain in the stomach, with complete suppression of urine. Subsequently, sensations of numbness of the hands and feet caused much distress and anxiety. In a period of twenty-four hours only about half an ounce of dark-colored urine could be obtained, containing blood-corpuscles and brownish tube-casts, and the presence of methemoglobin was shown with the spectroscope. The collapse increased, and death occurred in two days, preceded by jaundice.

The spleen, liver and kidneys were brown in color; the uriniferous tubules were filled with brownish masses. The red-blood corpuscles were changed in shape and appearance. A similar appearance after diphtheria may be due to the remedy and not the disease. Dr. Bohn condemns the delivery of chlorate of potassium into unprofessional hands, or its common sale as a harmless remedy.—*Medical Times.*

**Doctors in the West.**—The following statistics from the *Chicago Tribune* indicate that the West is even more overcrowded than the East :

| | Population. | Doctors. |
|---|---|---|
| Illinois | 3,077,871 | 5,899 |
| Indiana | 1.978,301 | 4,903 |
| Iowa | 1,624,615 | 3,035 |
| Kansas | 996,096 | 1,664 |
| Michigan | 1,636,937 | 2,924 |
| Minnesota | 780,773 | 914 |
| Nebraska | 452,403 | 807 |
| Nevada | 62,266 | 134 |
| Wisconsin | 1,315,407 | 1,548 |
| Dakota | 135,·77 | 212 |
| Montana | 39,159 | 77 |
| Idaho | 32,610 | 51 |
| Wyoming | 20,789 | 30 |
| Colorado | 194,327 | 570 |
| Missouri | 2,168,380 | 4 550 |
| Totals | 14,515,200 | 27,700 |

| | Population. | Doctors. | One to |
|---|---|---|---|
| Chicago | 503,185 | 918 | 548 |
| Denver | 35,629 | 137 | 160 |
| Detroit | 116,340 | 248 | 469 |
| Indianapolis | 68,538 | 264 | 259 |
| Kansas City | 55,785 | 167 | 332 |
| Milwaukee | 115,587 | 141 | 819 |
| Minneapolis | 46,887 | 121 | 387 |
| St. Louis | 350,518 | 338 | 474 |
| St. Paul | 41,473 | 75 | 553 |

**The Diagnosis of Sciatica.**—A diagnostic point in sciatica is given by De Beurmann which we have never seen aluded to. The patient lying on his back with the muscles of the leg and back relaxed, the affected leg is raised while in complete extension and flexed upon the abdomen. This causes marked pain in the course of the sciatic, especially intense at the sciatic notch, and the movement is resisted. If, then, the limb be lowered, and while the leg is flexed on the thigh the latter is again carried up on to the pelvis, no pain will be felt. This phenomenon depends on the fact, verified by De Beurmann in experiments on the cadaver, that great tension of the sciatic is exerted by flexion of the thigh when the leg is extended, but almost none when the leg is flexed.—*Boston Med. and Sur. Journal.*

**Erigeron Canadensis.**—In medicine, the volatile oil of erigeron is employed as an astringent to restrain passive hemorrhages from different parts of the body. It exerts a favorable influence in all passive hemorrhages, whether they come from the uterus, bladder, lungs or intestines. From one to five drops of the oil may be given at a dose, and repeated every one, two or three hours, if required. It may be dropped on lumps of sugar to administer, or it may be put up in capsules, or prepared in the shape of an emulsion, with sugar, gum arabic and water. Erigeron ls a valusble remedy as a stimulating hemostatic.—*Amer. Med. Record.*

**Cholera and the Migration of Birds.**—The *Med. Record* tells us that the fact that the swallows, which migrated from Marseilles at the outbreak of the pestilence, have not yet returned, and that there are no sparrows in the city, is adduced as evidence that the atmosphere is still vitiated. This migration of the birds has made a deep impression upon the public mind, and has led to a demand for a purification of the atmosphere by means of bonfires.

**The Song of the Tubercle Bacillus.**—Translated from the German of the Wiener Caricaturen :

Owner of the strongest glasses,
And the sharpest eyes to pore;
Famous chief, enough your triumph—
Koch, what would you with us more?

In the lungs we dwelt in safety
Till you came with cruel lore,
Stained us blue and magnified us—
Koch, what would you with us more?

You have planted us and bred us
In a dog! Oh! trial sore!
Robbed our nature of each secret—
Koch, what would you with us more?

We repent us of our evil,
Bitter tears our sad eyes pour;
Self-consumed, we die of phthisis—
Koch, what would you with us more?

—*The Medical Age.*

